# Longer diagnosis‐to‐ablation time is associated with recurrence of atrial fibrillation after catheter ablation—Systematic review and meta‐analysis

**DOI:** 10.1002/joa3.12294

**Published:** 2019-12-27

**Authors:** Raymond Pranata, Veresa Chintya, Sunu B. Raharjo, Muhammad Yamin, Yoga Yuniadi

**Affiliations:** ^1^ Faculty of Medicine Universitas Pelita Harapan Tangerang Indonesia; ^2^ Faculty of Medicine Universitas Kristen Krida Wacana Jakarta Indonesia; ^3^ Department of Cardiology and Vascular Medicine Faculty of Medicine Universitas Indonesia National Cardiovascular Center Harapan Kita Jakarta Indonesia; ^4^ Division of Cardiology Department of Internal Medicine Faculty of Medicine Universitas Indonesia Cipto Mangunkusumo National General Hospital Jakarta Indonesia

**Keywords:** atrial fibrillation, atrial fibrillation recurrence, catheter ablation, diagnosis‐to‐ablation time, time‐to‐ablation

## Abstract

**Background:**

Diagnosis‐to‐ablation time (DTAT) has been postulated to be one of the predictors of atrial fibrillation (AF) recurrence, and it is a “modifiable” risk factor unlike that of many electrocardiographic or echocardiographic parameters. This development may change our consideration for ablation. In this systematic review and meta‐analysis, we aim to analyze the latest evidence on the importance of DTAT and whether they predict the AF recurrence after catheter ablation.

**Methods:**

We performed a comprehensive search on topics that assess diagnosis‐to‐ablation time (DTAT) and AF recurrence from inception up until August 2019 through PubMed, EuropePMC, Cochrane Central Database, and http://ClinicalTrials.gov.

**Results:**

There was a total of 3548 patients from six studies. Longer DTAT was associated with increased risk for AF recurrence in all studies included. Meta‐analysis of these studies showed that DTAT had a hazard ratio (HR) of 1.19 [1.02, 1.39], *P* = .03; *I* ^2^: 92% for AF recurrence. Upon sensitivity analysis by removing a study, HR became 1.24 [1.16, 1.32], *P* < .001; *I* ^2^: 29%. Meta‐analysis on DTAT time >3 years had HR 1.73 [1.54, 1.93], *P* < .001; *I* ^2^: 45% for the recurrence of AF. Upon subgroup analysis of data that compared >6 years to <1 year, the HR was 1.93 [1.62, 2.29], *P* < .001; *I* ^2^: 0%.

**Conclusion:**

Longer DTAT time is associated with an increased risk of AF recurrence. Hence, determining management at the earliest possible moment to avoid delay is of utmost importance.

## Graphical abstract

Diagnosis‐to‐ablation time >3 years is associated with the increased risk of atrial fibrillation recurrence after catheter ablation.

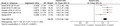

## 1 INTRODUCTION

Atrial fibrillation (AF) is the most prevalent cardiac arrhythmia worldwide.^1^ Despite advances in catheter ablation technology, the recurrence for AF is high, ranging from 46.2% to 60.0% in a single‐procedure ablation in a 2013 meta‐analysis.^2^ The number of recurrences is expected to decrease with new methods of ablation.^3^ Nevertheless, effort to predict the recurrence of AF is an exciting development.^4^, ^5^, ^6^, ^7^ It enables physicians to stratify the risk of AF recurrence and weight the risk and benefit on the patients before performing catheter ablation.

Recently, longer diagnosis‐to‐ablation time (DTAT) has been postulated to be one of the predictors of AF recurrence.^8^ Diagnosis‐to‐time ablation is a “modifiable” risk factor unlike to that of many electrocardiographic or echocardiographic parameters; this risk factor can be minimized. This development is exciting because it may change our consideration for ablation. In this systematic review and meta‐analysis, we will analyze the latest evidence on the importance of DTAT and whether they predict the AF recurrence after catheter ablation. To the best of our knowledge, this is the first meta‐analysis on the DTAT and its impacts on AF recurrence after catheter ablation.

## 2 METHODS

### 2.1 Search strategy

**Figure 1:**
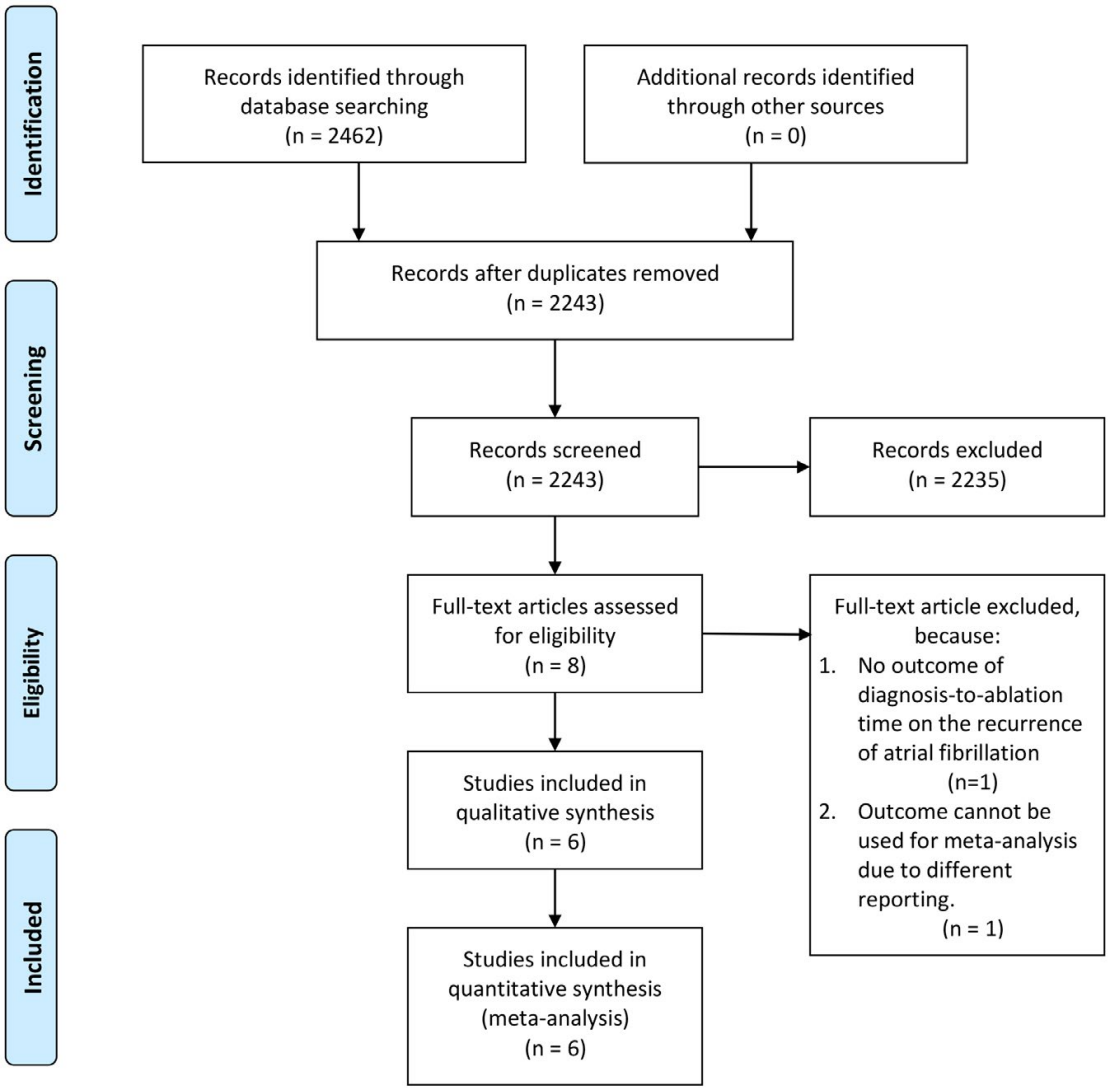
Study flow diagram

We performed a comprehensive and systematic literature search on topics that assesses DTAT and AF recurrence with keywords [“diagnosis to ablation time”, “atrial fibrillation”, and “recurrence”] and its synonym from inception up until August 2019 through PubMed, EuropePMC, Cochrane Central Database, http://ClinicalTrials.gov and hand‐sampling from potential articles cited by other studies. The records were then systematically evaluated using inclusion and exclusion criteria. We also perform hand‐sampling from references of the included studies. Two researchers (R.P and V.C) independently performed an initial search, discrepancies were resolved by discussion. A Preferred Reporting Items for Systematic Reviews and Meta‐Analyses flowchart of the literature search strategy of studies is presented in Figure 1.

### 2.2 Selection criteria

The inclusion criteria for this study are all studies that assess the DTAT and AF recurrence. We include all related clinical researches/original articles and exclude case reports, and review articles.

### 2.3 Data extraction

Data extraction and quality assessment were done by two independent authors (R.P. and V.C.) using standardized extraction form which includes authors, year of publication, study design, sample size, patient characteristics, persistent/paroxysmal AF, follow‐up period, DTAT, and atrial arrhythmia recurrence (AF/atrial flutter [AFL]/atrial tachycardia [AT]).

### 2.4 Statistical analysis

To perform the meta‐analysis, we used RevMan version 5.3 software (Cochrane Collaboration). We used the hazard ratio (HR) and a 95% confidence interval. We used mean difference and its standard deviation as a pooled measure for the continuous data. Inconsistency index (*I* ^2^) test, which ranges from 0% to 100%, was used to assess heterogeneity across studies. A value above 50% or *P* < .05 indicates statistically significant heterogeneity. We used the Inverse Variance method for HR with a fixed‐effect model for meta‐analysis, and a random‐effect model was used in case of heterogeneity. All *P* values were two‐tailed with a statistical significance set at .05 or below.

## 3 RESULTS

We found a total of 2462 results. We screened 2243 records after removing duplicates. Eight were relevant titles/abstract. After assessing eight full‐text for eligibility, we excluded two because of (a) no outcome of DTAT on the recurrence of AF and (b) outcome cannot be used for meta‐analysis due to different reporting. We included six studies in the qualitative synthesis and six studies in meta‐analysis (Figure 1; Table 1).^8^, ^9^, ^10^, ^11^, ^12^, ^13^ Five studies were prospective cohorts, and one study was a retrospective cohort. There was a total of 3548 patients from six studies.

**Table 1:** Summary of the included studies

Author	Study design	Sample (n)	Patients characteristics	Diagnosis of first AF episode	Primary outcome (recurrence/freedom)	Definition of recurrence	RF as ablation energy (%)	Paroxysmal AF (%)	Age, years (mean ± SD)	Male (%)	Follow‐up (mean)
Kawaji 2019	Restrospective Cohort	1206	First‐time RFCA for AF	Unclear; Possibly history taking and medical record	AF/AT with a blanking period of 90 d; without AAD	AF/AT > 30 s or requiring repeat ablation procedures	100	70.7	64.3 ± 9.5	71	5.0 ± 2.5 y
Bisbal 2019	Prospective Multicenter Cohort	309	First ablation of symptomatic drug‐refractory paroxysmal and persistent AF	History taking and medical record	AF/AFL with no blanking period; regardless of AAD	AF/AFL > 30 s	68	66.8	56.9 ± 10.1	71	3 y
Su 2019	Prospective Cohort	282	RFCA for AF refractory to AADs	Electronic medical record	AF/AFL/AT with 3 mo blanking period; without AAD after blanking period	AF/AFL/AT > 30 s	100	65.25	65.39 ± 10.45	53.9	18 mo
Greef 2018	Prospective Cohort (Middelheim‐PVI Registry)	1000	Symptomatic, drug‐resistant recurrent AF with no or limited structural heart disease undergoing a first PVI	Review of database, looking at 12‐lead ECG, Holter or event recording	AF with 1 mo blanking period; without AAD	AF > 30 s	90.7	58.5	60 ± 10	72	5 y
Lunati 2018	Prospective Cohort	510	Paroxysmal AF who undergo CBA	History taking and medical record	AF with 90 d blanking period; regardless of AAD	AF > 30 s	0	100	59.1 ± 10.6	67.5	16.3 ± 8.5 mo
Hussein 2016	Prospective Cohort	1241	RFCA for recurrent symptomatic persistent AF	Review of the data registry and medical records, persistent AF without a preceding paroxysmal AF	AF/AFL/AT with 3 mo blanking period; without AAD after blanking period	AF/AFL/AT > 30 s	100	0	61.0 ± 10.2	78.5	2 y

Abbreviations: AADs, antiarrhythmic drugs; AF, atrial fibrillation; AFL, atrial flutter; AT, atrial tachycardia; CBA, cryoballoon ablation; ECG, electrocardiography; PVI, pulmonary vein isolation; RF, radiofrequency; RFCA, radiofrequency catheter ablation.

### 3.1 Study characteristics

Most of the study enlisted first‐time ablation of AF. Three studies used radiofrequency catheter ablation, one study used cryoballoon ablation, and the other two studies are included both. The DTAT was defined as first episode of AF, which was mostly based on review of registry data or medical record to the time of catheter ablation. The primary outcome of the studies was either freedom/recurrence of atrial tachyarrhythmia (AF/AFL/AT) with a blanking period ranging from 0 to 3 months. The definition of recurrence varied between studies that includes AF, AFL, and/or AT greater than 30 seconds. The patients were mixed of paroxysmal and persistent AF, except in two studies which exclusively enrolled paroxysmal AF or persistent AF, respectively. The mean age was in the range of 50‐60 years old and male is more than female in all studies, and most of them had a 70% male. The mean follow‐up ranges from 16 months to 5 years (Table 1). The ablation time was not affected by DTAT, meta‐analysis of ablation time from two studies by Lunati et al and Bisbal et al demonstrated no significant difference between longer and shorter DTAT.

### 3.2 DTAT and AF recurrence

**Figure 2:**
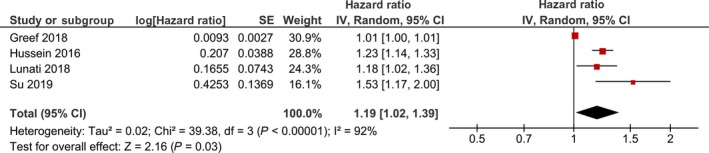
Diagnosis‐to‐ablation time and atrial fibrillation recurrence. Forest‐plot showing that lengthier diagnosis‐to‐ablation time is associated with the increased risk of atrial fibrillation recurrence after catheter ablation

Longer DTAT was associated with increased risk for AF recurrence in all studies included. Meta‐analysis of these studies showed that DTAT had a HR of 1.19 [1.02, 1.39], *P* = .03; *I* ^2^: 92%, *P* < .001 for AF recurrence (Figure 2). Upon sensitivity analysis by removing Greef et al study, HR became 1.24 [1.16, 1.32], *P* < .001; *I* ^2^: 29%, *P* = .24. Meta‐analysis showed that DTAT time >1 year were associated with HR 1.60 [1.15, 2.23], *P* = .005; *I* ^2^: 0%, *P* = .45 for the recurrence of AF.

### 3.3 DTAT >3 years and AF recurrence

**Figure 3:**
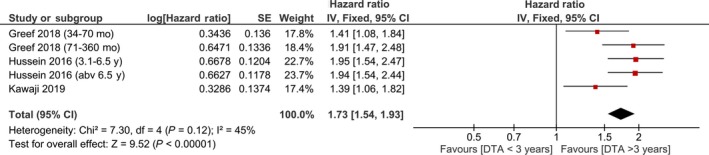
Diagnosis‐to‐ablation time >3 y and atrial fibrillation recurrence. Forest‐plot showing that diagnosis‐to‐ablation time >3 y is associated with the increased risk of atrial fibrillation recurrence after catheter ablation

Meta‐analysis showed that DTAT time >3 years were associated with HR 1.73 [1.54, 1.93], *P* < .001; *I* ^2^: 45%, *P* = .12 for the recurrence of AF (Figure 3). These studies compared >3 years DTAT with <1 year DTAT, except in a study by Kawaji et al who compared <3 years vs >3 years. Upon removal of Kawaji et al study the result become more significant with HR 1.81 [1.60, 2.05], *P* < .001; *I* ^2^: 30%, *P* = .23. Upon subgroup analysis of data that compared >6 years to <1 year, the HR was 1.93 [1.62, 2.29], *P* < .001; *I* ^2^: 0%, *P* = .93.

## 4 DISCUSSION

This systematic review and meta‐analysis concluded that a longer DTAT is associated with AF recurrence after ablation as early as 12 and 36 months have been demonstrated to have a profound effect on the AF recurrence. Interestingly, shorter DTAT is shown to have a higher AF freedom in both paroxysmal and persistent AF, as shown by studies by Hussein et al who included 100% persistent AF samples and Lunati et al which included a 100% paroxysmal AF samples.^10^, ^12^ Paroxysmal AF occasionally (10%‐20%) progress to persistent AF and probably, considering the delay in that 10%‐20% patients, DTAT is still a significant predictor of AF recurrence.^14^ Kawaji et al stated that the risk for ischemic stroke and transient ischemic attack increased with DTAT, half of the events occurred after the diagnosis of AF, and it was found that the prevalence of stroke is higher in those with DTAT >3 years compared to <3 years, this means that DTAT also possibly affect cardiovascular outcome.^11^ Kawaji et al also showed the rate of heart failure hospitalizations was significantly lower in those with short DTAT further emphasizing the importance of DTAT in outcomes other than AF recurrence.^11^ The cause of heterogeneity in the DTAT >3 years is Kawaji et al study which compared <3 years vs >3 years DTAT, as opposed to the other study included the meta‐analysis which compared >3 years with <1 year DTAT; however, the results did not differ much upon removal of the study on sensitivity analysis. The rate of complications on longer vs shorter DTAT were not sufficiently reported, there was a study that showed no significant difference on the rate of complication in both groups.^12^

Atrial fibrillation causes electrical and structural remodeling in the atrium, which further provides substrate, promote occurrence, and maintenance of AF and worsen the condition in a vicious cycle manner.^15^ AF has a progressive nature and the complexity increases over time; hence, a longer DTAT means a lengthier period in which the atrium is in the aforementioned vicious cycle.^16^ Theoretically, longer DTAT will have a more severe atrial remodeling compared to shorter DTAT and has a higher chance of AF recurrence after conversion to sinus rhythm.

### 4.1 Clinical implication

The clinical implication of this finding might be extensive, although further studies should be conducted before integrating this finding to routine clinical practice. There are many electrocardiographic and echocardiographic predictors; however, not much can be done to reduce the risk for AF recurrence in these patients. Diagnosis‐to‐time ablation is a modifiable risk factor that is dependent on the physician and the patient's agreement. Early ablation is associated with a better AF freedom, however, to translate this to clinical practice, we suggest trials to perform ablation in within 1‐3 years of AF diagnosis vs rhythm/rate control and also measures outcome such as major adverse cardiovascular events, mortality, safety, and quality of life. Such trials will enable us to determine extensively whether more aggressive treatment is needed and may have the potential to change the landscape of AF treatment in the future.

### 4.2 Study limitation

Limitation of this systematic review includes publication bias in which DTAT might not be reported by studies if it is not significant. The data regarding other outcomes based on DTAT were reported with a different format across the studies, that it was not possible to perform a meta‐analysis on the outcome. Due to the limited number of studies and unavailability of data, the authors were unable to perform subgroup analysis or meta‐regression to determine whether DTAT will affect paroxysmal and persistent AF differently.

## 5 CONCLUSION

Longer DTAT time is associated with an increased risk of AF recurrence. Hence, determining management at the earliest possible moment to avoid delay is of utmost importance. Currently, there are studies that indicate rate and rhythm control had similar effectiveness; however, there are no trials that perform ablation in within 1‐3 years of AF diagnosis vs rhythm/rate control. We suggest that further trials perform ablation on patients within 1‐3 years of AF diagnosis, taking rate and/or rhythm as the control group.

## CONFLICT OF INTEREST

The authors declare no conflict of interests for this article.

## AUTHORS CONTRIBUTION

Raymond Pranata conceived and designed the study and drafted the manuscript. Raymond Pranata and Veresa Chintya acquired the data and drafted the manuscript. Raymond Pranata, Veresa Chintya, and Yoga Yuniadi interpreted the data and performed extensive research for the manuscript. Muhammad Yamin and Yoga Yuniadi performed critical revision to the manuscript. Raymond Pranata analyzed the data statistically. All authors contributed to the writing of manuscript.
